# The binding characteristics of isoniazid with copper–zinc superoxide dismutase and its effect on enzymatic activity

**DOI:** 10.1186/1752-153X-7-97

**Published:** 2013-06-06

**Authors:** Nana Du, Liangquan Sheng, Zhaodi Liu, Xiaojuan Hu, Huajie Xu, Shuisheng Chen

**Affiliations:** 1 College of Chemistry and Chemical Engineering, Fuyang Normal College, Fuyang 236041, People’s Republic of China; 2 College of Chemistry and Chemical Engineering, Anhui University, Hefei 230039, People’s Republic of China

**Keywords:** Superoxide dismutase, Isoniazid, Interaction, Antioxidation

## Abstract

**Background:**

Isoniazid (INH) is front-line anti-tuberculosis (TB) drugs, which are usually prescribed to TB patients for a total period of 6 months. Antituberculosis drug-induced hepatotoxicity (ATDH) is a serious adverse reaction of TB treatment. It is reported that INH-induced hepatotoxicity is associated with oxidative stress. Superoxide dismutase (SOD, EC 1.15.1.1) is the key enzyme for the protection of oxidative stress, which catalyzes the removal of superoxide radical anion, thereby raising the need to better understand the interaction between INH and SOD.

**Results:**

The experimental results showed that the fluorescence intensity of Cu/Zn-SOD regularly decreased owing to form a 1:1 INH-SOD complex. According to the corresponding association constants (*K*_SV_) between INH and SOD obtained from Stern–Volmer plot, it is shown that values of *K*_A_ are 1.01 × 10^4^, 5.31 × 10^3^, 3.33 × 10^3^, 2.20 × 10^3^ L · mol^−1^ at four different temperatures, respectively. The binding constants, binding sites and the corresponding thermodynamic parameters (^Δ^*H*, ^Δ^*G* and ^Δ^*S*) were calculated. A value of 3.93 nm for the average distance between INH and chromophore of Cu/Zn-SOD was derived from Förster theory of non-radiation energy transfer. The conformational investigation showed that the presence of INH resulted in the microenvironment and conformational changes of Cu/Zn-SOD. In addition, Effects of INH on superoxide dismutase activity was examined.

**Conclusions:**

The results show that the hydrogen bonding and van der Waals forces play major roles in stabilizing the 1:1 INH-SOD complex. After addition of INH during the range of the experiment, the conformation and microenvironment of Cu/Zn-SOD are changed, but the activity of Cu/Zn-SOD is not changed.

## Background

Tuberculosis (TB) is one of the leading causes of death due to a single disease, accounting for up to 2 million lives each year [1]. Isoniazid (INH) is the foremost first-line antibiotic used to treat TB, which continues to be the cornerstone of all antituberculosis regimens and remains the only agent recommended for tuberculosis chemoprophylaxis for children [2].

Despite numerous and intensive studies, we have a limited knowledge of the action mechanism of INH [3-6]. The consensus opinion is that in the presence of a slow flux of H_2_O_2_ or superoxide, KatG converts INH into a radical species which is subsequently coupled to NADH to form an INH-NAD(P) adduct. The INH-NAD adduct is a potent inhibitor of *Mtb* InhA, an enoyl reductase required for the elongation steps in mycolic acid biosynthesis [7,8]. It has been recently suggested that the superoxide reactivity affects antitubercular activity of INH to some extent, and thereby raises the needs to better understand the interaction between INH and superoxide [9].

Furthermore, with increasing occurrence of TB all over the world, a growing number of patients may be at risk for severe adverse drug reactions (ADRs) such as antituberculosis drug-induced hepatotoxicity (ATDH) when treated with antituberculosis chemotherapy [10,11]. ATDH can be fatal if it is not recognised at an early stage, after which therapy should be interrupted timely. Moreover, ATDH has a negative impact on therapy adherence, decreases success rates of treatment and eventually increases treatment failure, relapse or the emergence of drug resistance. The occurrence of hepatotoxicity related to INH has been well-defined [12] and shown to increase as a result of drug-drug pharmacokinetic or pharmacodynamic interactions [13]. It is generally acknowledged that INH-induced hepatotoxicity is associated with oxidative stress [14].

Superoxide dismutase (SOD, EC 1.15.1.1) is an antioxidant enzyme in animals, plants, fungi and bacteria, which catalyzes the removal of superoxide radical anion (O_2_^−^•) to hydrogen peroxide (H_2_O_2_) that can be subsequently converted to water by the enzyme catalase [15,16]. Therefore, it is the key enzyme for the protection of oxidative stress and could be a suitable candidate drug to protect liver from ATDH [17-19]. To better understand the protective mechanism of SOD, the interaction between SOD and INH should be investigated.

Protein–drug interaction is a hot topic in fields of medicine, chemistry and biology. Also, due to the effects on the drug of pharmacokinetics when bounded to protein, there is an increasing interest in protein from clinical and pharmaceutical perspectives [20-22]. Furthermore, the binding of drugs to protein in vitro is considered as a model in protein chemistry to study the binding behavior of protein. Consequently, the investigation of the binding between drugs and SOD is of fundamental importance in pharmacology and pharmacodynamics. In addition, the analysis of the interaction between SOD and small drug molecules is interesting to some extent, because the binding of specific small molecules to SOD has only been characterized in detail for a few examples [23,24].

In this paper, the interaction between INH and Cu/Zn-SOD has been investigated with fluorescence and ultraviolet spectroscopy. The binding mechanism, binding constants, binding sites and binding distance were obtained. The nature of the binding force was analyzed based on the thermodynamic parameters. Also, the effects of INH on the conformation of Cu/Zn-SOD were examined by synchronous fluorescence and three-dimensional fluorescence spectroscopy. What is more, the effects of INH on the activity of Cu/Zn-SOD were also explored. We hope that this work can provide useful information for pharmacology of INH.

## Results and discussion

### Effects of INH on Cu/Zn-SOD fluorescence

**Figure 1:**
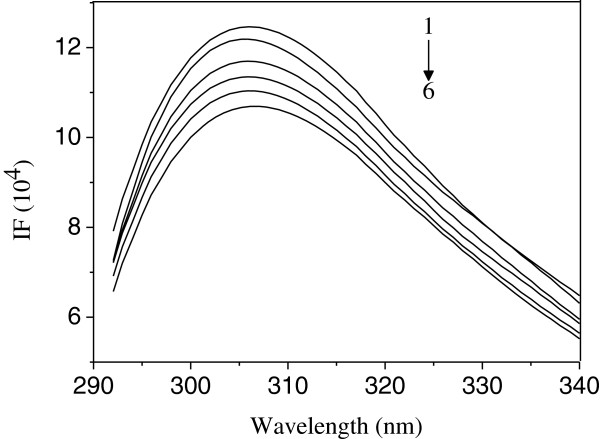
**Fluorescence spectra of SOD in the presence of various concentrations INH at 299 K (pH = 7.4, λ**_**ex **_**= 280 nm).***c*(SOD) = 4.0 × 10^−6^ mol · L^−1^; *c*(INH)/(10^−5^ mol · L^−1^), 1–6: 0, 0.8, 1.6, 2.4, 3.2, 4.0, respectively.

The fluorescence of Cu/Zn-SOD comes from the tryptophan, tyrosine and phenylalanine residues [25]. With 280 nm of excited wavelength, the maximum emission peak of Cu/Zn-SOD could be achieved at 306 nm [26]. Figure 1 shows the fluorescence spectra of Cu/Zn-SOD upon the addition of INH. Obviously, with the increasing concentration of INH, the fluorescence intensity decreased regularly but without significant position-shift. The strong quenching of the fluorescence clearly indicated that the interaction between INH and Cu/Zn-SOD changed the microenvironment of chromophore or the tertiary structure of Cu/Zn-SOD.

### The quenching mechanism of Cu/Zn-SOD fluorescence by INH

Fluorescence quenching refers to any processes which decrease the fluorescence intensity of a sample such as excited state reactions, energy transfers, ground-state complexes formation and collisional process [27]. Mechanisms of quenching are usually divided into dynamic quenching and static quenching [27]. Different quenching mechanisms can be distinguished by their different dependence on temperature and excited-state life time. As higher temperatures could result in larger diffusion coefficients, the dynamic quenching constants are expected to be larger with an increasing temperature. In contrast, the increase of temperature is likely to result in a decreased stability of complexes; thus, the values of static quenching constants are expected to be smaller. In the present work, the fluorescence-quenching spectra of Cu/Zn-SOD with presence of different concentrations of INH at four different temperatures (the temperatures used were 292, 295, 299 and 303 K) were measured. The fluorescence quenching was usually analyzed by the well-known Stern–Volmer equation [28,29]:

(1) $$F_{0}/F=1+K_{\mathrm{SV}}\left[Q\right]=1+K_{q}\tau_{0}\left[Q\right]$$

where *F*_0_ and *F* are fluorescence intensities of Cu/Zn-SOD in the absence and presence of the quencher (INH), respectively, *K*_q_ is the quenching rate constant, *K*_SV_ is the Stern–Volmer constant, *τ*_0_ is the average life time of the molecule without quencher and the fluorescence lifetime of the biopolymer is 10^−8^ s, [Q] is the quencher concentration.

**Figure 2:**
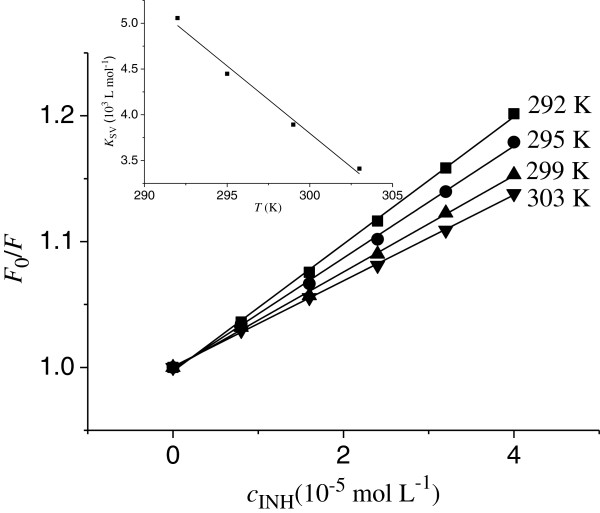
**Stern-Volmer plots for the quenching of SOD by INH at four different temperatures, (pH = 7.4).** Inset: *K*_SV_-T.

Figure 2 shows the Stern-Volmer plots of the Cu/Zn-SOD quenching fluorescence by INH. The values of *K*_SV_ obtained from the slop of plots are 5.06 × 10^3^, 4.45 × 10^3^, 3.89 × 10^3^ and 3.41 × 10^3^ L · mol^−1^, respectively. It is evident that the *K*_SV_ values are inversely correlated with temperature (inset in Figure 2), and *K*_q_ is much greater than the value of the maximum scatter collision quenching constant (2.0 × 10^10^ L · mol^−1^ · s^−1^), which indicates the static mechanism of quenching and reveals that the fluorescence quenching is caused by specific interaction and complex formation between Cu/Zn-SOD and INH molecules [27].

### Calculation of binding parameters

For static quenching, the following equation was employed to calculate the binding constant and binding sites [30,31]:

(2) $$\log\frac{F_{0}-F}{F}=\log K_{A}+n\log\left[Q\right]$$

where *F*_0_, *F* and [Q] are the same as in Eq.(1), *K*_A_ and *n* are the binding constant and the number of binding sites respectively, *K*_A_ and *n* can be obtained from the plots of log[(*F*_0_- *F*)/*F*] vs. log[Q] for the INH–SOD system at four temperatures. The calculated results are shown in Table 1. The results show that *K*_A_ decreases with the increase of temperature, which probably indicates the formation of a compound partially decomposes at higher temperatures. The value of *n* is equal to approximately 1, indicating the involvement of a single binding site in the INH–SOD interaction.

**Table 1:** Binding parameters, number of binding sites and thermodynamic parameters of INH–SOD at four temperatures

***T*(K)**	***K***_**SV **_**(L · mol**^**-1**^**)**	***K***_**A **_**(L · mol**^**-1**^**)**	***n***	**Δ *G *(kJ · mol**^**-1**^**)**	**Δ *H *(kJ · mol**^**-1**^**)**	**Δ *S *(J · mol**^**-1**^ **· K**^**-1**^**)**
292	5.06 × 10^3^	1.01 × 10^4^	1.069	−22.38 (s = 1.69)	−105.70 (s = 9.54)	−284.66 (s = 18.50)
295	4.45 × 10^3^	5.31 × 10^3^	1.019	−21.04 (s = 1.73)		−286.31 (s = 15.67)
299	3.89 × 10^3^	3.33 × 10^3^	0.995	−20.16 (s = 1.54)		−285.42 (s = 19.92)
303	3.41 × 10^3^	2.20 × 10^3^	0.999	−19.36 (s = 1.26)		−284.29 (s = 19.31)

**Figure 3:**
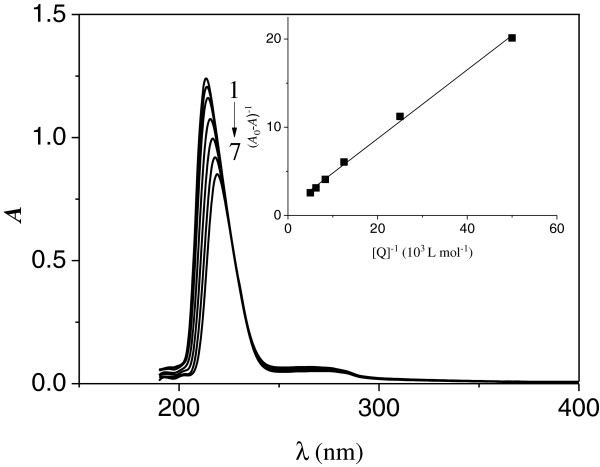
**UV-Vis absorption spectra of SOD in the absence and presence of INH system (pH = 7.4, T = 303 K).***c*(SOD) = 4.0 × 10^−6^ mol · L^−1^; *c*(INH)/(10^−5^ mol · L^−1^) , 1–7: 0, 2, 4, 8, 12, 16, 20, respectively. Insert: (*A*_0_- *A*) ^−1^ vs. [Q] ^−1^.

In order to confirm the probable quenching mechanism of Cu/Zn-SOD by INH, the absorption spectra of Cu/Zn-SOD in the presence and absence of INH at 303 K were recorded and presented in Figure 3. As seen in Figure 3, with the addition of INH the absorbance peak around 213 nm, which was mainly caused by the transition π-π* of peptide bonds in SOD decreased regularly with peak red-shift (from 213 to 220). Thus, the ground state complex between SOD and INH was possibly formed. The absorbance peak caused by the transition π-π* gets red-shifted with the increase of solvent polarity, so we can draw a conclusion that the polarity of the microenvironment of SOD is altered, leading to a conformational change that the peptide strands of SOD are extended and its hydrophobicity is decreased [28,30,31].

According to literature [32,33], when one small organic molecule binds to biological macromolecule, the relationship between absorbance intensity of biological macromolecules and the small organic molecule concentration can be written as follows:

(3) $${\left(A_{0}-A\right)}^{-1}=A_{0}^{-1}+K_{A}^{-1}A_{0}^{-1}{\left[Q\right]}^{-1}$$

where *A*_0_ and *A* are the absorbance intensities of Cu/Zn-SOD in the absence and presence of the INH, respectively, *K*_A_ and [Q] are the binding constant and INH concentration. As shown in the inset in Figure 3, plot of 1/(*A*_0_-*A*) against 1/[Q] gives a straight line, the correlation coefficient of which is 0.9986 and the binding constant (*K*_A_) is equal to 2.32 × 10^3^ L · mol^−1^. The results further prove that only one molecule of INH binds Cu/Zn-SOD.

### Thermodynamic analysis and intermolecular forces

The intermolecular forces of interaction between small molecules and biomolecules mainly include hydrogen bonding, van der Waals forces, electro static forces and hydrophobic forces. Within a small temperature range, the enthalpy of interaction can be regarded as a constant leading to the following equations [34,35]:

(4) $$\ln\left(K_{A2}/K_{A1}\right)=\mathrm{\Delta H}\left(1/T_{1}-1/T_{2}\right)/R$$

(5) ΔG=ΔH−TΔS

(6) $$\Delta S=-\left(\Delta G-\Delta H\right)/T$$

where *R* is the gas constant, *T* is the absolute temperature and *K*_A_ is the apparent binding constant at corresponding temperature *T*. If Δ*S* > 0 and Δ*H* > 0, the main force of interaction is the hydrophobic force, if Δ*S* > 0, Δ*H* < 0, the main force is electro static attraction, if Δ*S* < 0, Δ*H* < 0, the main force includes both van der Waals forces and hydrogen bonding. Table 1 shows the values of Δ*H*, Δ*S* and Δ*G* at four temperatures. The negative values of Δ*G* reveal that the interaction process is spontaneous. As Δ*S* < 0 and Δ*H* < 0, which indicates that the interaction between INH and Cu/Zn-SOD is mainly due to hydrogen bonding and van der Waals forces [34,35].

### Energy transfer between Cu/Zn-SOD and INH

**Figure 4:**
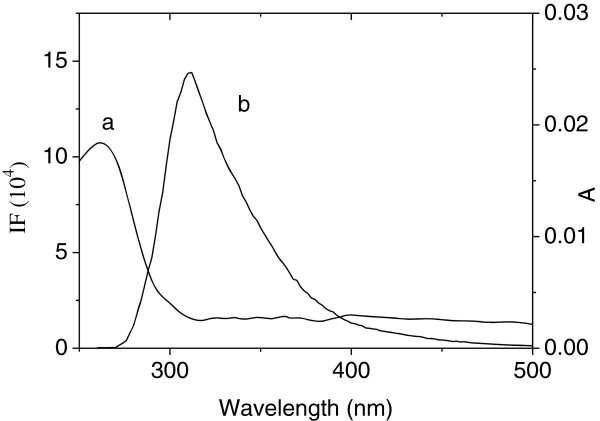
**Spectral overlap of UV-Vis absorption spectrum of INH (a) with the fluorescence emission spectrum of SOD (b).***c* (SOD) = c (INH) = 4.0 × 10^−6^ mol · L^−1^ (pH = 7.4, T = 303 K).

The energy transfer in biochemistry is important, because the efficiency of transfer can be used to evaluate the distance between the ligand and the chromophore in protein. The overlap of the UV–Vis absorption spectrum of INH with the fluorescence emission spectrum of Cu/Zn-SOD, as is shown in Figure 4. According to Förster nonradiative energy-transfer theory, the efficiency of energy transfer is *E*, the critical distance for 50% energy transfer is *R*_0_, and the actual distance of separation is *r*. These values were calculated by Eqs. (7, 8, 9) [35-37]:

(7) $$E=1-\frac{F}{F_{0}}=\frac{R_{0}^{6}}{R_{0}^{6}+r^{6}}$$

(8) $$R_{0}^{6}=8.8\times 10^{-25}K^{2}\Phi N^{-4}J$$

where *r* is the distance from the ligand to the chromophore of the protein, and *R*_0_ is the Förster critical distance at which 50% of the excitation energy is transferred to the acceptor.

(9) $$J=\frac{\sum F\left(\lambda \right)\epsilon \left(\lambda \right)\lambda^{4}\Delta \lambda }{\sum F\left(\lambda \right)\Delta \lambda }$$

where F(λ) is the fluorescence intensity of the donor at the wavelength range λ, and ϵ (λ) is the molar absorption coefficient of the acceptor at wavelength λ. In this case, *K*_2_ = 2/3, N = 1.336 and Φ = 0.13 [36]. According to the equation (7), (8), (9), we could calculate that *J* = 2.3 × 10^−13^ cm^3^ · L · mol^−1^, *E* = 0.022, *R*_0_ = 2.58 nm, and *r* = 3.93 nm. The binding distance between INH and Cu/Zn-SOD is shorter than 7 nm, which indicates that the energy transfer from Cu/Zn-SOD to INH occurs with high possibility [35-37]. It is also suggested that the binding of INH to Cu/Zn-SOD is conducted through energy transfer, which is consistent with a static quenching mechanism.

### Conformation investigation of Cu/Zn-SOD

**Figure 5:**
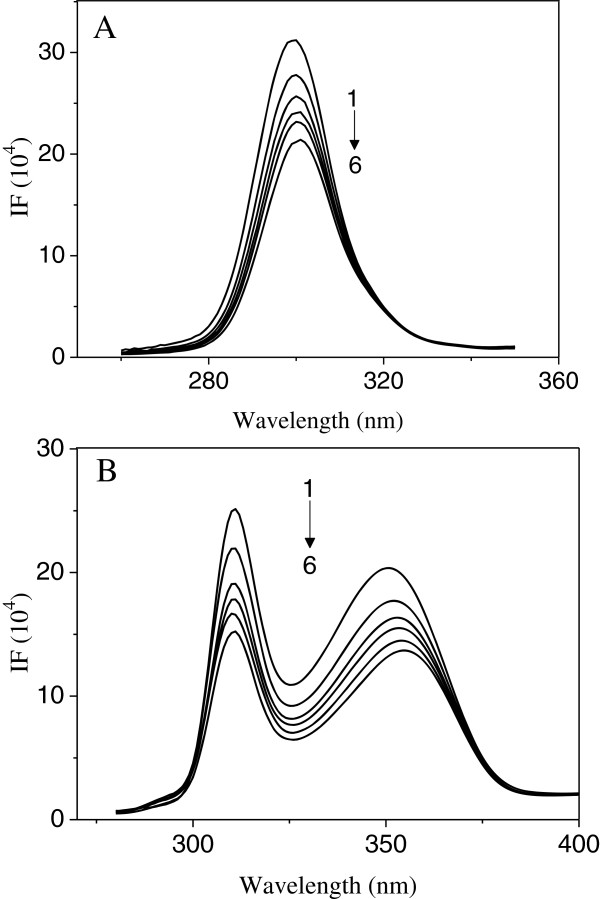
**Synchronous fluorescence spectra of SOD (pH = 7.4, T = 299 K). A** Δλ = 20 nm, **B** Δλ = 80 nm. *c* (SOD) = 4.0 × 10^−6^ mol · L^−1^; c (INH)/(10^−5^ mol · L^−1^) 1–6: 0, 4, 6, 8, 10, 12, respectively.

The influence of INH on conformational changes of Cu/Zn-SOD was assessed by synchronous fluorescence. Synchronous fluorescence measurement provides information about the molecular microenvironment in the vicinity of the fluorophore functional groups [38]. When the wavelength shift (Δλ) between the emission and excitation wavelengths was stabilized at 20 or 80 nm [39], the synchronous fluorescence gives the characteristic information of tyrosine or tryptophan residues, respectively. Figure 5A and Figure 5B show the effects of INH on the synchronous fluorescence of Cu/Zn-SOD. As seen in Figure 5, the fluorescence intensities of tryptophan residue and tyrosine residue decreased with the increase of the concentration of INH. The maximum emission wavelength of tyrosine residues does not undergo a significant shift in the investigated concentration range. In contrast, an obvious red shift was observed for tryptophan residues, which indicated that the polarity around tryptophan residues increased. In other words, tryptophan residues were placed in a less hydrophobic environment and were more exposed to the solvent.

**Figure 6:**
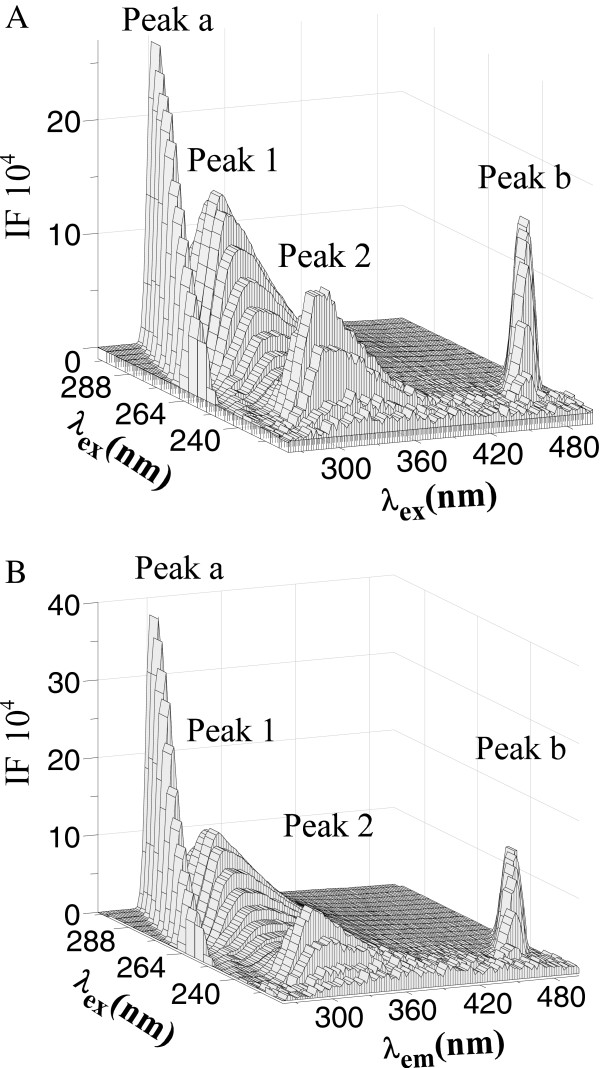
**Three-dimensional fluorescence spectra of SOD (A) and the INH–SOD system (B) (pH = 7.4, T = 299 K). A***c* (SOD) = 4.0 × 10^−6^ mol · L^−1^, *c* (INH) = 0; **B***c* (SOD) = 4.0 × 10^−6^ mol · L^−1^, *c* (INH) = 4.0 × 10^−5^ mol · L^−1^.

In recent years, the three-dimensional fluorescence technique has been widely used, because it allows fluorescence characteristics to be acquired by changing the excitation and emission wavelengths simultaneously. We studied the conformational and microenvironmental changes of Cu/Zn-SOD by comparing the spectral changes in the absence and presence of INH, as shown in Figure 6. In these figures, peak a is the Rayleigh scattering peak (λ_ex_ = λ_em_), the peak intensity of which increased with the addition of INH. A reasonable explanation may be that an INH-SOD complex formed after the addition of INH, increased the diameter of the macromolecule, and thus enhanced the scattering effects [40]. Peak b is the second-ordered scattering peak (λ_em_ = 2λ_ex_). Peak 1 (λ_ex_ = 280.0 nm, λ_em_ = 306.0 nm) mainly reveals the spectral behavior of the Trp and Tyr residues since excitation of SOD at this wavelength mainly reveals their intrinsic fluorescence, and the fluorescence of the phenylalanine residue can be negligible [25]. Besides peak 1, there is another strong fluorescence peak (peak 2, λ_ex_ = 232.0 nm, λ_em_ = 306.0 nm), which mainly exhibits the fluorescence spectral behavior of polypeptide backbone structures [40]. Both peaks were involved in fluorescence quenching of Cu/Zn-SOD by INH to different degrees. By analyzing the intensity changes of peak 1 and peak 2, in the absence and presence of INH, the fluorescence intensity ratio of peak 1 to peak 2 is 1.16:1 and 1.18:1, respectively. All of these phenomena and analyses revealed that the binding of INH to SOD induced some microenvironmental and conformational changes in Cu/Zn-SOD.

### Effects of INH on Cu/Zn-SOD activity

**Figure 7:**
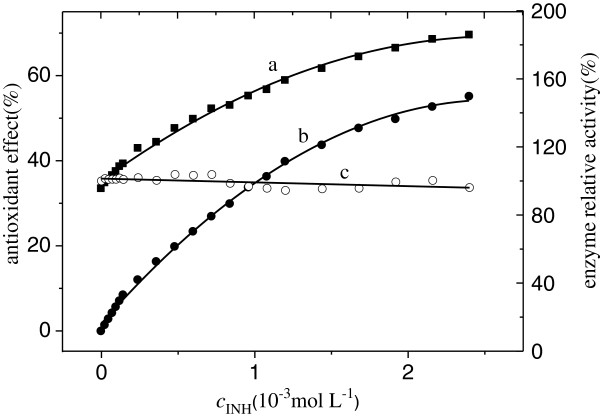
**Effect of INH on the activity of superoxide dismutase (SOD) (pH = 7.4, T = 303 K). a** antioxidant effect of INH-SOD, **b** antioxidant effect of INH, c enzyme relative activity of SOD.

The activity of Cu/Zn-SOD after the addition of INH was studied to investigate the effects of INH on superoxide dismutase activity. Figure 7a shows the relationship between antioxidant effect of INH-SOD and INH concentration. Figure 7b shows the relationship between antioxidant effect of dissociative INH and its concentration. Due to antioxidant effect of INH in the range of the experiment, the antioxidation of INH-SOD was strengthened with increasing INH concentration. The enzyme activity was determined by subtracting the antioxidant effect of INH from antioxidant effect of INH-SOD (Figure 7c). The results show that the enzyme activity is almost the same throughout the experiment. The observation leads to the conclusion that the INH does not affect the activity of Cu/Zn-SOD.

### Experimental

#### Materials

Cu/Zn-SOD was purchased from Doulai Biotechnology Company (Nanjing, China), INH was purchased from Taizhou Medical Corporation (Zhejiang, China). SOD was directly dissolved in redistilled water to prepare the stock solution (0.12 mM), and the stock solution was kept in the dark at 0–4°C. INH solution was obtained by dissolving it in redistilled water (1.2 mM). 0.05 M Tris-HCl buffer solution of pH 7.4 and 8.2 were prepared. Other reagents are local products of analytical grade. The water used was redistilled and ion-free.

Fluorescence measurements were carried out in 1.0 cm quartz cells on a FluoroMax-4 spectrophotometer (Horiba, Japan) equipped with a thermostat bath. UV–vis spectra were recorded on an 8000A spectrophotometer (Beijing Purkinje General Corporation, China). All pH measurements were made using a pHS-3 digital pH-meter (Chengdu Sanke Instrument Corporation, China) with a combined glass electrode.

#### Assay

A 3.0 mL Tris–HCl (pH 7.4, 0.05 M) buffer solution containing 4 μM SOD was titrated by successive additions of 1.2 mM INH solution and the concentration of INH varied from 0 to 40 μM. Titrations were done manually by using the microinjectors. After reaction for 10 min, fluorescence spectra were measured in the range of 290–450 nm at the excitation wavelength of 280 nm. The fluorescence spectra were performed at four temperatures (292, 295, 299 and 303 K). The excitation and emission slits were 5 nm.

The synchronous fluorescence spectra were recorded at 299 K from 260 nm to 350 nm (for tyrosine residues) at Δλ = 20 and from 280 to 400 at Δλ = 80 nm (for tryptophan residues). The excitation and emission slits were10 nm.

Three-dimensional fluorescence spectra of SOD were recorded at 299 K in the presence and absence of INH with an excitation wavelength in the range 230–300 nm and an emission wavelength in the range 260–500 nm. The excitation and emission slits were 5 nm.

A 3.0 mL Tris–HCl (pH 7.4, 0.05 M) buffer solution containing 4 μM SOD was titrated by successive additions of 1.2 mM INH solution and the concentration of INH varied from 20 to 200 μM. Titrations were done manually by using the microinjectors. After reaction for 10 min at 303 K, absorption spectra of SOD-INH were recorded in the range 190–400 nm.

The activity of Cu/Zn-SOD was assayed by using the pyrogallol autoxidation method. The assay mixture contained 5.0 ml Tris–HCl (pH 8.2, 0.05 M) buffer, 0.1 mM pyrogallol solution, 2.5 nM SOD solution and different concentrations of INH. The concentration of INH varied from 0 to 2.4 mM. Absorption at 325 nm at 303 K against time was recorded using a spectrophotometer. The SOD activity was determined as pyrogallol autoxidation rate.

## Conclusions

In this paper, the interaction between INH and SOD was studied using fluorescence and ultraviolet spectroscopy at different temperatures under imitated physiological conditions. The results show that the quenching mechanism of fluorescence of SOD by INH is a static quenching process. The binding constants, binding sites and the corresponding thermodynamic parameters (Δ*H*, Δ*G* and Δ*S*) were determined, which indicates that hydrogen bonding and van der Waals forces play a major role in the binding process. According to Förster theory of nonradiation energy transfer, the binding distance between Cu/Zn-SOD and INH is 3.93 nm and the energy transfer occurs between SOD and INH with high probability. UV–vis, synchronous fluorescence and three-dimensional fluorescence studies indicates that the interaction leads to a change in the conformation and microenvironment of Cu/Zn-SOD. Moreover, determination of SOD activity in different concentration INH leads to the conclusion that the binding of INH with SOD does not affect the activity of Cu/Zn-SOD. This report has special significance in pharmacology and clinical medicine as well as methodology.

## Competing interests

The authors declare that they have no competing interests.

## Authors’ contributions

ND made a significant contribution to acquisition of data, analysis and manuscript preparation. XH has made a substantial contribution to experimental design and data analysis. ZL and HX participated in partial experiments. LS made a significant contribution to experimental design, data analysis, and manuscript revision. SC participated in study design and manuscript revision. All authors read and approved the final manuscript.
